# Early Clinical Effects of Novel Partial D3/D2 Agonist Cariprazine in Schizophrenia Patients With Predominantly Negative Symptoms (Open-Label, Non-controlled Study)

**DOI:** 10.3389/fpsyt.2021.770592

**Published:** 2022-01-24

**Authors:** Stanislav V. Ivanov, Anatoly B. Smulevich, Evgeniya I. Voronova, Kausar K. Yakhin, Tangyul Z. Beybalaeva, Alena A. Katok

**Affiliations:** ^1^Department of “Borderline” Mental Pathology and Psychosomatic Disorders, Federal State Budgetary Scientific Institution Mental Health Research Center, Moscow, Russia; ^2^Department of Psychiatry and Psychosomatic Medicine, Federal State Autonomous Educational Institution of Higher Education I.M. Sechenov First Moscow State Medical University of the Ministry of Healthcare of the Russian Federation (Sechenovskiy University), Moscow, Russia; ^3^Department of Psychiatry, Kazan State Medical University, Kazan, Russia

**Keywords:** schizophrenia, negative symptoms, pharmacotherapy, clinical effects, cariprazine

## Abstract

**Background:**

Because of limited efficacy of antipsychotics against negative symptoms in schizophrenia new drugs with wider spectrums of clinical efficacy are very desirable. The newer 3rd generation antipsychotic cariprazine presents the unique mode of action acting as partial agonist predominantly for dopamine D3- and in lesser extent D2-receptors. Cariprazine is found to be effective in the treatment of negative symptoms in schizophrenia comparing to second generation antipsychotic risperidone.

**Objectives:**

To evaluate initial effects of cariprazine in schizophrenia patients with predominantly negative symptoms.

**Design and Patients:**

Open-label, non-controlled study included 60 adult schizophrenia patients (F20 on ICD-10, 49% males) with predominantly negative symptoms (Positive and Negative Syndrome Scale, S factor score for negative and positive symptoms, PANSS-FSNS ≥ 15 and PANSS-FSPS <19) treated with cariprazine (starting daily dose 1.5 mg followed by upward titration by 1.5 mg weekly up to 6 mg if needed) were assessed with PANSS, CAINS (The Clinical Assessment Interview for Negative Symptoms), CDSS (Calgary Depression Scale for Schizophrenia), and SAS (Simpson-Angus Scale for Extrapyramidal Symptoms) scales at baseline and on week 1, 2, and 4.

**Results:**

Most patients (75%) improved during 28 days of cariprazine treatment. At the end of assessment (day 28) mean starting total scores for negative symptoms on PANSS-NS and CAINS scales significantly (*p* < 0.05) reduced by 4.3 and 4.9, respectively, with no significant changes in depression symptoms (CDSS). Cariprazine tolerability was very good, only four patients discontinued because of TEAEs (akathisia, insomnia).

**Conclusions:**

The results of this study suggest early effect of cariprazine on negative symptoms at least in some schizophrenia patients with predominantly negative symptoms starting from 1 to 2 weeks of treatment and could be useful for determination of early clinical predictors for efficacy. Considering limitations of open-label design with no control groups these data need to be confirmed.

## Introduction

Negative symptoms are regarded as a key factor affecting both the clinical and social prognosis of the schizophrenia, more important than positive symptoms (1, 2). Despite certain achievements in improving the efficacy and safety of treatments for schizophrenia driven by the development of atypical antipsychotics, pharmacological management of negative symptoms remains a challenge that, in contrast to treating positive and affective disturbances, is beyond the capacity of most available methods of pharmacotherapy (3, 4).

To date, the most convincing and promising data in the context of pharmacological management of negative symptoms have been obtained in clinical trials of a novel third-generation antipsychotic cariprazine. Cariprazine belongs to the class of partial dopamine agonists and has a unique 10-fold greater affinity for D3 receptors than for D2 receptors (unlike other third-generation drugs aripiprazole and brexpiprazole) (5, 6). Cariprazine is approved in Europe and the Russian Federation as an acute and maintenance treatment for schizophrenia in adult patients when given at a daily dose of 1.5–6 mg.

According to available publications, cariprazine is the only antipsychotic drug with a proven effect on primary negative symptoms that is verified according to the current methodological requirements (4, 7). The evidence for the efficacy of cariprazine in the treatment of negative symptoms was obtained in a 26-week, randomized, double-blind, comparative (with no placebo control) study of cariprazine 4.5 mg/day and risperidone 4 mg/day in 460 adult patients with predominant and persistent negative symptoms (8). The statistically significant superiority of cariprazine over risperidone in reducing negative symptoms (PANSS negative symptom factor score) was observed from week 14 of therapy, and it increased steadily until the end of treatment. Cariprazine has been shown to have a superior effect on the majority (five out of seven) of negative symptoms according to the PANSS scale score, including blunted affect, emotional withdrawal, passive/apathetic social withdrawal, poor rapport, difficulty in abstract thinking (9).

However, according to published data, differences between cariprazine and risperidone in terms of the change from baseline in PANSS negative symptom subscale scores over time were observed as early as at week 1 of therapy. Moreover the differences between the two treatments showed further steady growth, although not being statistically significant up to week 14 of therapy (8, 9). Hence, it may be assumed that cariprazine exerts its initial therapeutic effect on negative symptoms from the first 1-2 weeks of therapy. If this hypothesis is confirmed, this opens up perspectives for further identification of significant early clinical predictors of the efficacy of cariprazine in the first weeks of treatment that correlate with the long-term treatment prognosis in patients with schizophrenia with predominantly negative symptoms.

To confirm this assumption, this study was conducted to assess the initial effect of cariprazine at standard doses (1.5–6 mg/day) during the first 4 weeks of treatment in patients with schizophrenia with predominantly negative symptoms.

## Study Design and Patients

The study was conducted at the clinic of Department of Borderline Mental Pathology and Psychosomatic Disorders of the Federal State Budgetary Scientific Institution “Mental Health Research Center (director—professor T. P. Klyushnik) and by employees of the Department of Psychiatry of FSBEI HE “Kazan State Medical University” of the Ministry of Health of the Russian Federation (head of the department—prof. K. K. Yakhin) at clinical departments of the National Autonomous Healthcare Institution “Bekhterev Republican Clinical Psychiatric Hospital” of the Ministry of Health of the Russian Federation (chief medical officer—F. G. Ziganshin)^1^. In accordance with the objective of the study, the sample included 60 inpatients (31 females, 29 males; average age 35.6 ± 9.1 years) with a confirmed ICD-10 diagnosis of schizophrenia; socio-demographic characteristics of the sample are presented in Table 1.

**Table 1:** Socio-demographic characteristics of the study sample (*n* = 60).

**Parameter**	**No. of patients**
	**Abs. (%)**
**Gender**	
Males	29 (49%)
Females	31 (51%)
**Education**	
Higher/incomplete higher	40 (66%)
Secondary specialized	11(18.3%)
**Social status**	
Unemployed	47 (78.3%)
Receiving disability assistance	35 (58%)
**Family status**	
Married	10 (16%)
Divorced	5 (9%)
Single	45 (35%)

The study sample included patients diagnosed with schizophrenia (F20). The reported duration of the disease ranged from to 2 to 26 years and the number of episodes in the medical history was 2 to over 10.

Patients were sequentially and continuously enrolled in the study upon their admission to the clinical departments in accordance with the inclusion criteria, either immediately or after stabilization if hospitalization was associated with disease exacerbation. The study required four visits to the clinic: 1 day before the start of treatment with cariprazine, on day 7 of cariprazine treatment, on day 14 of cariprazine treatment, and on day 28 of cariprazine treatment.

During the study, psychopathological and psychometric methods were used: PANSS (Positive and Negative Syndrome Scale) (10); CAINS (The Clinical Assessment Interview for Negative Symptoms) (11), CDSS (Calgary Depression Scale for Schizophrenia) (12), CGI-I (Clinical Global Impression—Improvement) (13). To assess the tolerability throughout study treatment, adverse events leading to dose modification or discontinuation of cariprazine were recorded, as well as the change of the SAS score (Simpson-Angus Scale for Extrapyramidal Symptoms) over time (14).

Inclusion criteria were as follows: disease duration ≥ 2 years; stable state with no psychotic episodes during ≥6 months before enrollment; prevalence of negative symptoms with minimal/no positive symptoms during ≥6 months as per the clinician's assessment and in line with the following PANSS criteria: PANSS negative symptom factor score (PANSS-FSNS) ≥ 15; score ≥ 4 for at least two negative symptoms (blunted affect, passive or apathetic social withdrawal, lack of spontaneity and flow of conversation); informed consent obtained.

Exclusion criteria were as follows: pronounced positive symptoms as per the clinician's assessment and in line with the following PANSS criteria: PANSS positive symptom factor score (PANSS-FSPS) ≥ 19; moderate/severe depressive disorders as per the clinician's assessment and the CDSS criterion: total score > 6; clinically significant parkinsonism as per the clinician's assessment and the SAS criterion: total score for the first eight items > 3; organic CNS disorders; alcohol and/or psychoactive substances abuse/dependence.

After initial screening and eligibility assessment against the inclusion criteria, patients received cariprazine (Reagila) once daily, with or without food, for 28 days at the following daily doses: starting dose of 1.5 mg once daily with up-titration by 1.5 mg per week up to 6 mg once daily according to criteria as follows: (1) no changes in negative symptoms according to investigator's assessment and PANSS negative and CAINS scores; (2) no clinically significant adverse events (investigator's assessment and SAS scores).

A patient's state over time was assessed based on the change from baseline in PANSS negative symptom subscale scores as well as CAINS scores (total score and scores for individual items of the scale).

Given the resistance of negative symptoms and short duration of the assessment period, a reduction in baseline total scores on each of the specified scales at least of 3 points after 4 weeks of treatment plus ≤ 3 points (minimal or more pronounced improvement) on the CGI-improvement scale was chosen as an outcome measure.

Patients were withdrawn from the study in case of the following: individual drug intolerance or severe side effects, patient's refusal to continue treatment.

The statistical analysis was performed using the Microsoft Excel and STATISTICA v.12.5 software with the Wilcoxon *T*-test. A *p* ≤ 0.05 value was taken as the threshold level of statistical significance for comparisons between initial and each subsequent weekly assessment.

## Results

Of 60 patients enrolled in this study, 49 (81.7%) completed the planned 28-day treatment period. Hence, 11 (18.3%) patients prematurely dropped out from the study at week 2–3 of treatment. None of these premature discontinuations was related to safety concerns, i.e., the occurrence of potentially life-threatening adverse effects. All 11 patients discontinued treatment based on their own initiative; 4 (6.7 %) due to poor tolerability (see details in the discussion of treatment tolerability) while the remaining 7 (11.6 %) motivated their refusal to continue treatment with cariprazine by anxiety and agitation (week 1–2 of treatment, doses of 1.5–3 mg/day), yet not accompanied by exacerbation or relapse of psychotic disorders. Even though in all of these seven cases the clinician deemed the exacerbation of the mentioned symptoms to be mild/moderate, acceptable at the initial stage of therapy with a non-sedative antipsychotic and manageable with the use of anxiolytics and sedatives, cariprazine treatment was discontinued.

According to the method of therapy, all patients started treatment with cariprazine at a recommended initial dose of 1.5 mg/day followed by up-titration or down-titration due to lack of efficacy or side effects, respectively. Administration at the initial dose of 1.5 mg/day did not lead to a desirable effect. By week 4 of follow-up, the majority of patients (42 out of 49) had the optimal balance between therapeutic effect and tolerability in the dose range of 3 to 4.5 mg/day (Table 2).

**Table 2:** Patient disposition by cariprazine daily dose at each of 4 weeks of treatment.

**Cariprazine dose (mg/day)**	**Treatment week**			
	**1**	**2**	**3**	**4**
1.5	60	5	0	0
3	0	55	28	22
4.5	0	0	26	20
6	0	0	0	7
Total^*^	60	60	53	49

* *A decrease in the number of patients by week 4 is associated with premature treatment discontinuation in several cases*.

The prevalence of negative symptoms is supported by the ratio of mean baseline total scores on PANSS positive and negative syndromes subscales: 12.5 and 24.8, respectively, composite index minus 12.2 (Table 3). The mean baseline total CAINS score of 41.8 also reflects quite pronounced negative symptoms (given that the maximum score on this scale is 52) (Table 3).

**Table 3:** Changes in baseline scores for positive, negative, and extrapyramidal symptoms during 28 days of treatment with cariprazine (1.5–6 mg/day).

**Scale**	**Days of therapy**			
	**0**	**7**	**14**	**28**
PANSS negative	24.8	23.9	22.6	20.5*
PANSS positive	12.5	12.4	11.8	10.2
CAINS total	41.8	39.6	38.3	36.9**
SAS total	1.3	1.8	2.4	2.1

PANSS, Positive and Negative Syndrome Scale; CAINS, The Clinical Assessment Interview for Negative Symptoms; SAS, Simpson-Angus Scale for Extrapyramidal Symptoms; Significant changes from baseline values: *p < 0.05; **p < 0.01.

According to the accepted outcome measure (a reduction in baseline total scores for negative symptoms on the PANSS and CAINS scales by ≥3 points plus ≤ 3 points on the CGI-S scale), in a subgroup of 49 patients who completed 28 days of treatment with cariprazine, the therapy was considered effective in 45 patients and ineffective in 4 patients. Thus, 75% of patients from the overall sample (60 patients) met the efficacy criterion.

During treatment, there was a progressive weekly improvement in baseline PANSS scores at all stages of the assessment with concurrent reduction of positive and negative symptoms. However, it should be noted that at the end of week 4 the decrease in the baseline total negative symptom subscale score was more pronounced—by 4.3 points (from 24.8 to 20.5; *p* < 0.01), whereas the baseline score for positive symptoms showed only a minimum change from 12.5 to 10.2.

Starting from day 7 of therapy, there was a gradual decrease in the scores for all seven negative symptom PANSS items; the most pronounced changes over time were observed for such parameters as “emotional withdrawal” and “difficulty in abstract thinking,” for which the mean baseline scores decreased by 1.6 and 1.5 points, respectively (Table 4).

**Table 4:** Change from baseline in negative symptom scores for separate items of the Positive and Negative Syndrome Scale (PANSS) during the first four weeks of treatment with cariprazine.

**PANSS items for negative symptoms**	**Days of therapy**				**D0 vs. D28 difference**
	**0**	**7**	**14**	**28**	
Blunted affect	4.08	3.6	3.1	3.25	−0.83
Emotional withdrawal	4.5	3.1	2.8	2.9*	−1.6
Poor rapport	3.8	2.9	2.6	2.5	−1.3
Passive/apathetic social withdrawal	4.25	3.5	3.0	3.1	−1.15
Difficulty in abstract thinking	4.5	4.08	3.75	3.0*	−1.5
Lack of spontaneity and flow of conversation	3.75	3.8	3.6	3.25	−0.5
Stereotyped thinking	4.0	3.75	3.3	3.25	−0.75

*Significant changes from baseline values: *p < 0.05.

Changes from baseline in PANSS negative symptom subscale scores over time are consistent with changes in the CAINS scores (Table 3). There was also a significant and continuous decrease in the severity of negative symptoms across all stages of the assessment, with a reduction in the baseline score of 4.9 points (*p* < 0.01).

Patients with depressive symptoms were not included in the study in accordance with the inclusion criteria; this was verified using the Depression Scale for Schizophrenia (CDSS). Correspondingly, baseline CDSS scores were minimal, and the mean total score at cariprazine initiation in the study sample was close to zero and was equal to 2.3, well below the six-point threshold for depression (maximum total score on this scale is 27). However, there was a decrease in the baseline score to 1.2. after 4 weeks of therapy.

Cariprazine was well-tolerated. Only 4 out of 60 treatment discontinuations were associated with side effects. The main causes were severe akathisia and persistent insomnia, anxiety with a feeling of tension and irritability, which were reported in 3 patients during the first days of treatment at the initial dose of 1.5 mg/day and then got worse, and in another patient—at week 2 following up-titration to 3 mg/day. These events could not be adequately managed by dose reduction or use of anticholinergic and hypnotic drugs.

Extrapyramidal symptoms (EPS) were reported in 27 patients, mostly (*n* = 25) in the dose interval from 4.5 to 6 mg/day, including 2 of 4 patients who received 6 mg/day at week 4. Extrapyramidal symptoms were mild or moderate, did not require drug discontinuation, and were quickly resolved with minimal doses of anticholinergic drugs without reducing the daily dose of cariprazine.

The favorable tolerability profile is confirmed by a positive change over time in the SAS scores for abnormal movements (Table 3). The mean baseline score was 1.3, reflecting minimal movement disorders at the beginning of treatment with cariprazine. During therapy, there was only a slight increase in the baseline score of <2.5 points, and in the last week there was even a downward trend. Taking into account an up-titration of the dose at each subsequent week, and maximum daily dose of the drug (up to 6 mg/day) received by most patients at week 4, the values and changes over time in the SAS scores confirm low severity and ease of correction of the side effects of the drug.

### Clinical Case

A male patient, 37 years of age, single, incomplete higher education, 2nd degree disability due to a mental disorder. Diagnosis: shift-like schizophrenia. No family history of mental disorders. Pregnancy, delivery and early development with no particulars. The patient did not stand out among the other children, completed elementary school and 3 years (out of 5) at the university. He was anxious, impressionable, prone to avoidance behaviors, had several friends and was conform in companies. At the age of 19 (being a sophomore), he experienced the first psychotic episode with hallucinations and delusions and was treated at a residential psychiatric clinic, which resulted in the reduction of positive symptoms. Following the episode, he became passive, reserved and failed to study well. He left the university and worked irregularly, taking low-qualified jobs (delivery and cleaning services). Consequently, he had three more episodes with similar symptoms that required in-patient treatment; the last one was 6 years ago (at the age of 31). At that time the patient was given a disability group. No delusions or hallucinations have been noted during the last 5 years and the patient has remained stable. The patient was observed by a residential psychiatrist and received regular maintenance treatment with antipsychotic drugs (most recently olanzapine at the dose of 10 mg/day for 13 months). Due to the lack of positive dynamics and persistent functional decline, on relatives' advice the patient applied for hospitalization at the Federal State Budgetary Scientific institution “Mental Health Research Center.” The patient was eligible for this study of cariprazine and so took part in it. As the result of treatment, positive response was achieved at cariprazine dose 4.5 mg at week 3. The reduction in the baseline total negative symptom score on the PANSS scale was four points (from 22 to 18), and in the CAINS scores—four points. The patient became more active, did household chores without a reminder from his family, and did shopping on his own. He became more sociable, but only with his family. He began to show interest toward his old hobbies, started coin collecting again, attempted to play chess. With regard to side effects, the patient experienced insomnia at 3 mg/day, but when the dose was increased to 4.5 mg such events reduced.

## Discussion

According to currently available publications, this was the first study of initial effects of cariprazine in the treatment of patients with predominantly negative symptoms. Clearly, the open-label design, relatively short duration of treatment considering low sensitivity of negative symptoms to pharmacological therapy and a small number of observations do not provide sufficient validity and reliability of the obtained data. Hence, the results of the study should be interpreted with caution and may only be regarded as preliminary, with future verification needed.

However, the results of this study indicate a possible early response to treatment with cariprazine when given at standard doses (1.5–6 mg/day) starting from the first weeks of treatment in terms of positive dynamics of one's negative symptoms. Despite a relatively small change in the PANSS and CAINS negative symptom scores, which is understandable given the known resistance of negative symptoms to antipsychotics, the stable and progressive improvement in these scores and the achievement of statistical significance at the time of the final assessment is striking. It is also important to highlight the minimal severity and small changes in positive symptoms as well as the absence of clinically significant signs of depressive disorders and mild severity of Parkinsonism phenomena. Hence, it may be assumed that, as in a long-term comparative study of cariprazine and risperidone in the treatment of schizophrenia with predominantly negative symptoms, these data reflect a direct effect of therapy on primary negative symptoms already at the initiation of treatment and may be regarded as potential evidence of early effect.

These findings also suggest an activating effect of the drug when given at 1.5 mg/day (initial dose) and greater doses. This suggestion is supported by improved general and social activity, enhanced speech production and better social connection that were observed during treatment and confirmed by the change in the corresponding psychometric parameters over time. Insomnia, increased anxiety and tension may be indirect signs of activation. These observations are consistent with the tolerability profile of cariprazine established in clinical trials, in which insomnia is one of the most common side effects, no signs of severe sedation, and only minimal (rated as the best among antipsychotics) indicators of sleepiness are present (15).

The favorable tolerability profile with only mild EPS and akathisia being the most common and, apparently, problematic effect of the drug, is also consistent with the data of clinical studies (16).

Thus, the results of this 28-week open-label study suggest that a rapid initial treatment effect of cariprazine at doses of 3–6 mg/day with respect to deficit disorders in schizophrenic patients with predominantly negative symptoms is possible. The presented results require further verification and may be taken into account in the design of future studies, including those aimed at identifying early predictors of the therapeutic effect of cariprazine in reducing negative symptoms in schizophrenia.

## Data Availability Statement

The raw data supporting the conclusions of this article will be made available by the authors, without undue reservation.

## Ethics Statement

The studies involving human participants were reviewed and approved by Ethics Committee of Federal State Budgetary Scientific Institution Mental Health Research Center, Moscow, Russia. The patients/participants provided their written informed consent to participate in this study.

## Author Contributions

All authors listed have made a substantial, direct, and intellectual contribution to the work and approved it for publication.

## Conflict of Interest

The authors declare that the research was conducted in the absence of any commercial or financial relationships that could be construed as a potential conflict of interest.

## Publisher's Note

All claims expressed in this article are solely those of the authors and do not necessarily represent those of their affiliated organizations, or those of the publisher, the editors and the reviewers. Any product that may be evaluated in this article, or claim that may be made by its manufacturer, is not guaranteed or endorsed by the publisher.

## Footnotes

^1^The study was conducted in accordance with legal and ethical regulations of the Declaration of Helsinki, the current legislation of the Russian Federation and principles of Good Clinical Practice (GCP).
